# Impact of staff training on university productivity through job satisfaction: A study of ISO 9001- certified institutions

**DOI:** 10.1371/journal.pone.0306799

**Published:** 2024-07-09

**Authors:** Shahzaf Iqbal, Dr. Mubashir Hanif, Dr. Sohaib Khan

**Affiliations:** 1 School of Technology Management and Logistics, Universiti Utara Malaysia, Sintok, Kedah, Malaysia; 2 Professor Public Health, Health Services Academy, Islamabad, Pakistan; 3 Associate Professor Public Health, Health Services Academy, Islamabad, Pakistan; COMSATS University Islamabad - Wah Campus, PAKISTAN

## Abstract

The study examines the influence of staff training on university productivity through the job satisfaction of academic and administrative staff in ISO 9001-certified universities in Pakistan, utilizing a quantitative research approach. Data were gathered through online surveys using purposive sampling from academic and administrative staff, with analysis performed using SmartPLS-4. The results indicate that staff training significantly influences both job satisfaction and university productivity, with job satisfaction serving as a significant mediator. This research contributes to scholarly discourse by validating Organizational Learning Theory in ISO 9001-certified universities, highlighting the enhancement of productivity and job satisfaction through ISO 9001-aligned staff training. It also underscores the influence of QMS on employee attitudes and university productivity, highlighting the significance of ISO 9001 implementation, specifically through staff training, in university operations. Practical implications include recommendations for policymakers, administrators, and quality managers to prioritize ISO 9001-focused training to boost productivity and certification success, foster a culture of continuous learning, and improve educational quality and organizational outcomes. However, limitations such as the study’s cross-sectional design, purposive sampling, and focus on specific universities in Pakistan may limit generalizability. Future research should explore novel approaches to understanding factors influencing job satisfaction among academic and administrative staff to enhance productivity in ISO 9001-certified universities worldwide.

## 1. Introduction

Higher Education Institutions (HEIs) have undergone significant evolution from their traditional roles, gaining immense importance in the 21st century due to their diverse impacts on individuals, societies, and economies. Serving as hubs for knowledge, research, innovation, and the cultivation of global citizenship through entrepreneurship and collaboration, they play a pivotal role. Nevertheless, HEIs, particularly those in developing countries like Pakistan, face substantial challenges primarily due to inadequate funding. This deficiency compromises educational quality, hindering service quality, organizational productivity, and performance [1–4]. Pakistan allocated a mere 2.44% of its GDP to education in 2023 [5], exacerbating financial constraints for universities [6]. Thus, the imperative of efficient resource utilization becomes even more pronounced. Productivity, a critical determinant of organizational success, relies on the judicious allocation of resources, including raw materials, labor, skills, capital, infrastructure, intellectual property, managerial expertise, and financial backing, in the production of goods and services [7, 8].

With limited resources available for HEIs in Pakistan, establishing a robust quality management system (QMS) is crucial to enhance efficiency, reduce waste, and increase productivity. Quality education is pivotal for productivity improvement, equipping individuals with skills and knowledge that enhance efficiency, management effectiveness, product and process quality, technological advancements, regulatory compliance, and employee expertise [9, 10]. The adoption of ISO 9001 certification emerges as a notable framework capable of enhancing the operational efficiency and productivity of Pakistani universities. However, the significant disparity between ISO 9001 certification rates in educational institutions (25) compared to other sectors (2693) in Pakistan indicates a need for further adoption [11]. University leaders’ skepticism towards ISO 9001 contrasts with prior findings suggesting its potential to boost productivity through role definition, internal audits, work supervision, and planning facilitation [12]. Neglecting ISO 9001 adoption or implementation has resulted in subpar education quality and organizational inefficiencies, reflected in the scant representation of Pakistani universities in global rankings [13]. Addressing these challenges is crucial for enhancing academic quality, productivity, and university performance.

Investing in human capital through staff training initiatives is a promising strategy for addressing organizational challenges. Previous research emphasizes the significance of staff training in enhancing organizational performance by fostering improved skills, autonomy, job satisfaction, and positive management relationships [14, 15]. Additionally, scholars highlight training’s role in improving management systems, product quality, and various organizational factors, including employee retention and service quality [9, 16]. Research in higher education, exemplified by studies on Nigerian public universities [17] and ISO 9001 implementation [18], emphasizes the importance of continuous staff training. However, the relationship between staff training, job satisfaction, and university productivity remains underexplored, particularly in the context of Pakistani higher education.

### 1.1 Research gaps

This study identifies several critical gaps within the higher education context in Pakistan. Firstly, it highlights the issue of underfunding, which compromises educational quality and organizational productivity, exacerbated by Pakistan’s low GDP allocation to education. Secondly, it emphasizes the need for efficient resource utilization through the establishment of a robust QMS to enhance productivity. Thirdly, it points out the low adoption rates of ISO 9001 certification among educational institutions compared to other sectors, alongside university leaders’ skepticism towards ISO 9001 despite its proven potential to improve productivity. Additionally, the study notes a lack of research on the relationship between staff training, job satisfaction, and university productivity, particularly within the Pakistani context.

### 1.2 Significance and contributions of research

The current study presents significant theoretical and practical contributions. It validates the applicability of Organizational Learning Theory (OLT) in ISO 9001-certified universities, demonstrating a notable relationship between staff training aligned with ISO 9001 standards, job satisfaction, and university productivity. This research elucidates how ISO 9001 implementation impacts university operations, enhancing our understanding of quality management’s influence on productivity. Additionally, it provides insights into adapting these practices across diverse cultures. The study offers guidance for Pakistani universities on effective ISO 9001 implementation and informs human resource strategies by highlighting the benefits of systematic staff training. It emphasizes the importance of continuous learning and improvement within universities, offering administrators strategic initiatives to cultivate a culture of organizational learning. Finally, the findings assist policymakers and university administrators in shaping quality management policies in higher education, emphasizing the benefits of prioritizing staff training aligned with ISO 9001 standards for enhancing productivity, job satisfaction, and educational quality.

### 1.3 Research questions

To address the identified research gaps, the authors have formulated three research questions (RQs). The outcomes will benefit future researchers, along with academic and administrative staff in higher education globally, with a particular focus on Pakistan:

RQ1: What is the impact of staff training on job satisfaction and university productivity in the higher education context?
RQ2: What is the impact of job satisfaction on university productivity in the higher education context?
RQ3: Does job satisfaction mediate the relationship between staff training and university productivity in the higher education context?

The following sections of this article will delve into theoretical foundations, review pertinent literature, outline research methodology, present findings, and discuss implications for theory, practice, and future research, aiming to elucidate how staff training can catalyze the improvement of university productivity through job satisfaction of academic and administrative staff, thus fostering organizational excellence within Pakistan’s higher education landscape.

## 2. Literature review

### 2.1 Theoretical framework

The study employs the Organizational Learning Theory (OLT), which posits that organizations evolve, innovate, and enhance performance through knowledge acquisition, dissemination, and utilization over time [19]. The rationale for selecting OLT as the primary theory stems from its recognition of the critical role of staff training in organizational learning. Training serves as a primary mechanism for improving collective knowledge, skills, and competencies by facilitating knowledge acquisition, distribution, interpretation, and retention within the organization. Investing in training programs enhances individual employee capabilities and cultivates an adaptable and innovative workforce, thereby boosting overall performance and productivity.

OLT provides a comprehensive framework for evaluating university productivity by examining the impact of staff training (as an organizational learning process) on job satisfaction. This study aims to explore how universities enhance productivity through continuous learning initiatives such as training of academic and administrative staff and their job satisfaction. OLT emphasizes the significance of organizational structures, culture, and leadership in shaping learning processes, warranting an investigation into how staff training programs foster a culture of learning and promote knowledge acquisition, particularly in ISO 9001-certified universities.

**Fig 1:**
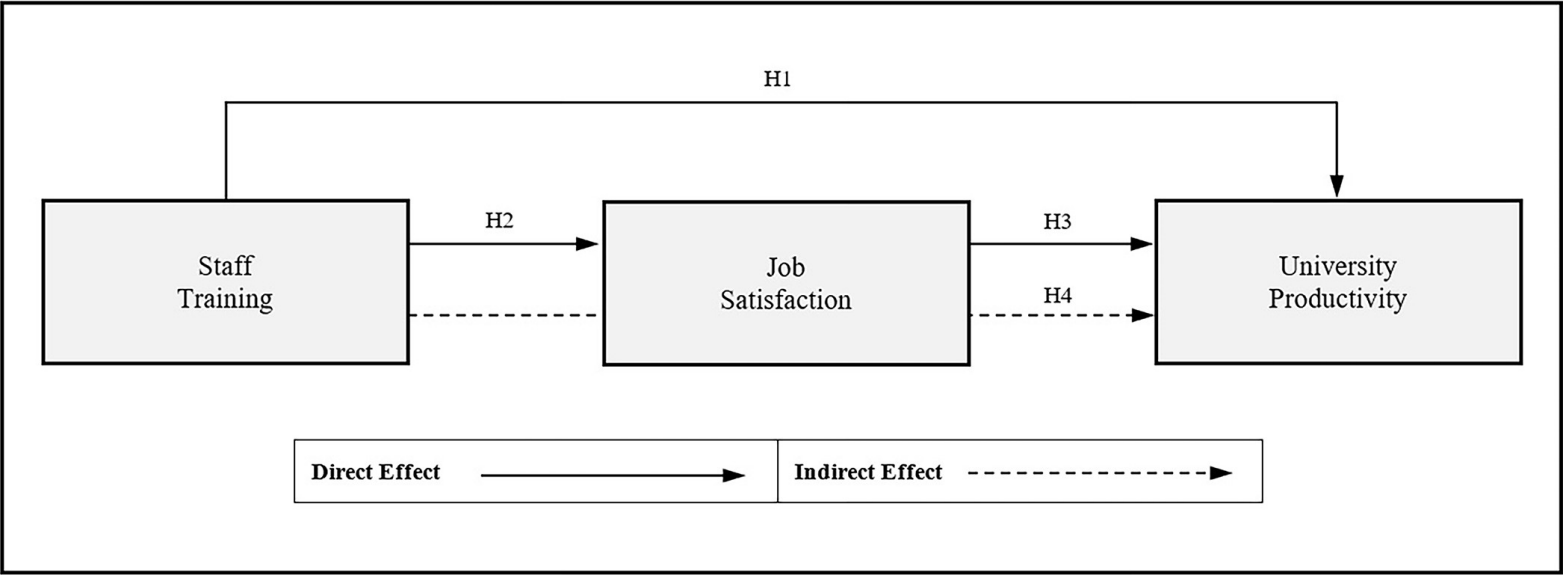
Theoretical framework.

Furthermore, OLT explores the enduring effects of learning on organizational performance and strategic capabilities, investigating how staff training initiatives strengthen organizational capacities, drive innovation, and enhance productivity over time. Given OLT’s alignment with quality management principles, it is relevant to explore the relationships among staff training, job satisfaction, and productivity within ISO 9001-certified universities. The theoretical framework adopts the principle of parsimony, emphasizing simplicity in explaining phenomena and proposing solutions, which is preferable to overly complex research frameworks that incorporate numerous factors [20]. This approach ensures clarity and precision in addressing the research questions. Fig 1 illustrates the theoretical framework, with staff training as the independent variable, job satisfaction as the mediator, and university productivity as the dependent variable.

### 2.2 University productivity

In today’s volatile economic landscape, organizational success is intricately linked to productivity [21], which is imperative for the survival of any company [22]. Before exploring productivity, it is essential to comprehend the concepts of effectiveness and efficiency, which constitute its foundation and are influenced by various factors [23]. Effectiveness entails meeting customer needs and expectations efficiently, while efficiency focuses on minimizing resource waste to achieve effectiveness [24]. These factors encompass management systems, product and process quality, technological advancements, and employee knowledge and skills [9]. While productivity and efficiency are often conflated, productivity involves effective resource utilization, whereas efficiency is gauged by performance [23]. Productivity serves as a pivotal metric for organizational success, signifying profitability and guiding employee recognition [8]. Furthermore, it propels economic growth, fosters innovation, and reduces waste [7].

In higher education, productivity entails efficiently allocating resources to achieve educational goals through effective teaching methods, optimized class time, high-quality publications, technological leverage for administrative efficiency, and ensuring student success via learning outcomes, graduation rates, and support services. Education is believed to enhance productivity by equipping individuals with skills and knowledge that amplify efficiency [10]. However, measuring productivity in academia is complex due to diverse activities such as teaching, research, administration, student achievement, resource management, and community engagement. Researchers have proposed methods for productivity improvement, such as increasing output, reducing input, or maintaining output levels with reduced input. Various factors influencing productivity encompass management effectiveness, product and process quality, technological advancements, regulatory influences, and employee knowledge and skills [9]. Nevertheless, productivity in education should align with institutional missions, particularly concerning graduates’ employability and career success [10].

### 2.3 Staff training and university productivity

Training academic and administrative staff on ISO 9001 requirements is essential for enhancing quality and improving university productivity, especially in regions like Pakistan where financial constraints are prevalent. Research indicates that trained employees demonstrate improved skills, autonomy, and job satisfaction, thereby fostering positive relationships with management [15]. Implementing ISO 9001 standards in universities requires comprehensive staff training to ensure compliance and efficient execution of QMS. While prior research has examined staff training’s impact on employee productivity in higher education settings, literature gaps persist regarding ISO 9001-related training within universities. For instance, it has been argued that the effectiveness of staff development programs in Nigerian public universities but did not address ISO 9001 training [17]. Similarly, another study surveyed faculty members in Kurdistan to assess the effects of staff training on productivity in teaching and administrative roles [25]. However, their findings lack generalizability due to limitations such as a small sample size and inadequate information on ISO 9001 certification among participating universities. Additionally, researchers examined the influence of staff training on librarians’ job performance, highlighting its positive impact on training initiatives [26]. Nonetheless, further investigation is required to grasp the complete influence of ISO 9001-related staff training on university productivity, encompassing insights from both faculty and quality managers. As a result, the following hypothesis is put forward:

**H1:** Staff training is significantly related to university productivity.

### 2.4 Staff training and job satisfaction

To enhance job satisfaction among employees in ISO 9001-certified universities, comprehensive training of faculty and administrative staff on QMS requirements is imperative. Such training is essential for ensuring compliance and effective QMS. By investing in training programs that empower employees, promote continuous improvement, and support professional development, universities can cultivate a proficient and motivated workforce. Numerous studies have highlighted the positive impact of training on job satisfaction. For instance, employee training contributes to both growth and satisfaction, as argued [16], while trained employees demonstrate enhanced skills and job satisfaction, fostering positive relationships with management [15]. Additionally, research conducted in Gauteng, South Africa, within the hospitality industry’s golf club sector, emphasized the significant influence of training on employee retention, development, and service quality [16]. Similarly, a study at Federal Polytechnic, Nasarawa, Nigeria, revealed that training significantly improved staff timeliness and work quality [27]. Moreover, supervisor support was found to significantly impact the job satisfaction of teachers in public high schools in Giresun province, Turkey [28]. Nevertheless, limited evidence exists regarding the link between staff training and job satisfaction in ISO 9001-certified Pakistani universities, necessitating further research. As a result, the following hypothesis is suggested:

**H2:** Staff training is significantly related to job satisfaction.

### 2.5 Job satisfaction and university productivity

Job satisfaction, a contemporary concept, contrasts historical practices where jobs were often inherited [29]. It is defined as the assessment of employee happiness at work [30], holding significance across various organizational settings, including higher education. Job satisfaction in higher education, encompassing fulfillment with various work aspects, is crucial for staff motivation, productivity, retention, and overall well-being, thereby influencing the quality of education and services. Factors shaping job satisfaction among academic staff include salary, job characteristics, work environment, conditions, and leadership dynamics [29]. While limited evidence previously existed regarding job satisfaction’s impact on university productivity, recent studies reveal its significant effects on employee performance [31] and work performance [32]. Similarly, a study among educators in public high schools across Turkey highlights the considerable influence of job satisfaction on job performance [28]. However, the narrow focus of these studies limits their generalizability, necessitating further empirical investigation within the higher education sector. Scholars have synthesized theories on job satisfaction within Pakistan’s higher education landscape, emphasizing its dynamic nature shaped by cultural factors. They argue that academic staff’s job satisfaction correlates with delivering quality education, enhancing student satisfaction, and improving university performance [30]. Nevertheless, the descriptive nature of this study and its reliance on a literature review pose limitations to its scope. Given the discussion and scant evidence on job satisfaction’s effect on university productivity in Pakistan, additional studies are imperative. Consequently, the following hypothesis is posited:

**H3:** Job satisfaction is significantly related to university productivity.

### 2.6 Job satisfaction as a mediator

Previous literature extensively explores job satisfaction, investigating its predictive value and its role as a mediator in various organizational contexts. It significantly influences organizational commitment [33], employee performance [31], and overall work performance [32]. Furthermore, job satisfaction serves as a mediator across various contexts. For example, it mediates between supervisor support and job performance [28], as well as among factors such as continuous learning, strategic leadership, and organizational commitment [33]. Additionally, it plays a mediating role between different leadership styles (e.g., transformational and transactional) and academic staff performance [34], and between organizational justice and citizenship behavior [35]. Nevertheless, a recent study conducted in State Polytechnic colleges in Indonesia presented divergent outcomes: although job satisfaction was found to significantly mediate the correlation between work environment and employee performance, it did not demonstrate such mediation concerning compensation and employee performance [36]. This discrepancy underscores the variability in the mediating role of job satisfaction and prompts a call for additional evidence, particularly within ISO 9001-certified universities in Pakistan. Thus, the following hypothesis is posited:

**H4:** Job satisfaction mediates the relationship between staff training and university productivity.

## 3. Methodology

### 3.1 Sample and data collection

The target population of this study comprised ISO 9001 certified universities in Pakistan. According to the latest ISO survey, only 25 institutions in Pakistan held ISO 9001 certification [11]. The unit of analysis was the institution, while the units of observation were academic and administrative staff. Purposive sampling was selected due to the absence of a sampling frame listing all 25 ISO 9001-certified institutions in Pakistan. This method was deemed appropriate as it allows for the selection of participants likely to provide relevant information [20]. Seven universities were identified through website searches as a result, employing a judgmental purposive sampling technique. Similar approaches have been used in prior studies under comparable conditions [37], justified by participants’ expertise in quality management within the education sector [38]. To mitigate potential biases inherent in non-probability sampling, criteria were established based on participant’s willingness to engage, familiarity with the subject matter, and extensive experience with ISO 9001 requirements through their universities’ implementation and maintenance of the standard. The recruitment spanned three months, from October 5, 2023, to December 31, 2023. A total of 200 online questionnaires were distributed to participants from the selected universities. All participants provided informed consent, guaranteeing confidentiality and non-disclosure of identifying information. From the distributed questionnaires, 135 responses were received. Thirteen responses were excluded due to consistent response patterns, resulting in 121 valid responses and a response rate of 60.5%. To confirm the adequacy and representativeness of the sample size, G*Power software was employed, based on two predictors, a statistical power of 0.95, a significance level of 0.05, and a moderate effect size of 0.15 [39–41]. The calculation indicated a minimum sample size requirement of 107. With an actual sample size of 121, our study surpasses this requirement, ensuring robust statistical power for analysis.

### 3.2 Measures

The current investigation employed an online questionnaire designed on a 5-point Likert scale, comprising 33 items divided into two sections. The initial segment addressed demographic information through seven questions, while the subsequent section consisted of 26 questions adapted from established literature to ensure content validity. Among these, 15 items focused on staff training [42], exemplified by a sample statement: “Training on ISO 9001 standard requirements emphasizes decreasing informal interactions in situations where formality is advantageous”. Additionally, five items addressed job satisfaction [43–45], represented by the statement, “I find my work to be very satisfying”. Furthermore, six items assessed university productivity [46–48], with a sample statement indicating, “Our university uses its technological resources efficiently”. This study obtained approval from the Ethics Committee of the Health Services Academy, Islamabad, Government of Pakistan (Ref. F. No. 20-10/2023-omi/HSA-007) on October 2, 2023. All participants, including academic and administrative staff, provided written informed consent, which assured confidentiality, data security, and non-disclosure of private information prior to completing the survey questionnaire. Moreover, an ethics statement presented at the beginning of the questionnaire underscored the voluntary nature of participation, with participants guaranteed data confidentiality. Before commencing the main study, a pre-test was administered to a panel of six experts comprising two senior professors, three quality directors, and one quality practitioner. Their profound expertise in pertinent domains yielded invaluable feedback for enhancing the questionnaire.

## 4. Data analysis and results

### 4.1 Demographic characteristics

Out of 121 respondents, 71.9% were from public sector universities, while 28.1% were from private sector universities. Geographically, 43% were from Islamabad, 28.1% from Karachi, and 14.9% each from Jamshoro and Lahore. Male respondents constituted 52.1%, with 47.9% being female. The majority fell within the 41–50 age bracket (50.4%), followed by 31–40 (46.3%). Predominant qualifications were Ph.D. (52.9%) and MSc (38%). Professional designations included assistant professors (32.2%), lecturers (25.6%), and associate professors (24%). Engineering accounted for the highest departmental representation at 35.5%, followed by social sciences (18.2%), computer sciences (17.4%), management sciences (16.5%), and Quality Enhancement Cell (QEC) (12.4%) (see Table 1).

**Table 1:** Respondents profile.

Items	N	%
Sector		
Public Sector	87	71.9
Private Sector	34	28.1
City		
Islamabad	52	43.0
Karachi	34	28.1
Jamshoro	18	14.9
Lahore	17	14.0
Gender		
Male	63	52.1
Female	58	47.9
Designation		
Professor	7	5.8
Associate Professor	29	24.0
Assistant Professor	39	32.2
Lecturer	31	25.6
Director	7	5.8
Deputy Director	4	3.3
Assistant Director	4	3.3
Age		
30 or less	3	2.5
31–40	56	46.3
41–50	61	50.4
51–60	1	0.8
Qualification		
BS/Master	3	2.5
MS/M.Phil	46	38.0
PhD.	64	52.9
Post. Doc.	8	6.6
Department		
Social Sciences	22	18.2
Management Sciences	20	16.5
Computer Sciences	21	17.4
Engineering	43	35.5
QEC	15	12.4

Source: Authors’ own findings.

### 4.2 Descriptive statistics

The descriptive statistics in Table 2 present mean (M) and standard deviation (SD) values for all three variables: staff training (M = 3.59, SD = 0.58), job satisfaction (M = 3.38, SD = 0.48), and university productivity (M = 3.59, SD = 0.66). The results indicate that staff training and university productivity share the same mean score, but the standard deviation of university productivity is slightly higher than that of staff training. Job satisfaction has the lowest mean score but also the smallest standard deviation. Overall, standard deviation values for all variables range from 0.48 to 0.66, suggesting a closely clustered distribution around the mean.

**Table 2:** Descriptive statistics.

	N	Minimum	Maximum	Mean	Std. Deviation
Staff Training	121	2.07	4.87	3.5917	0.58313
Job Satisfaction	121	2.20	4.40	3.3802	0.48125
University Productivity	121	1.83	5.00	3.5909	0.65687

Source: Authors’ own findings.

### 4.3 Multicollinearity and common-method bias (CMB)

Before hypothesis testing, the data underwent analysis using SmartPLS-4 software to detect potential multicollinearity and common-method bias (CMB). Results showed that all “Variance Inflation Factor” (VIF) values for each construct/variable were below 5 [49], indicating no multicollinearity. Additionally, barring one exception, all VIF values remained below the recommended threshold of 3.3 [50], providing further validation of the absence of both CMB and multicollinearity.

### 4.4 Measurement model

**Fig 2:**
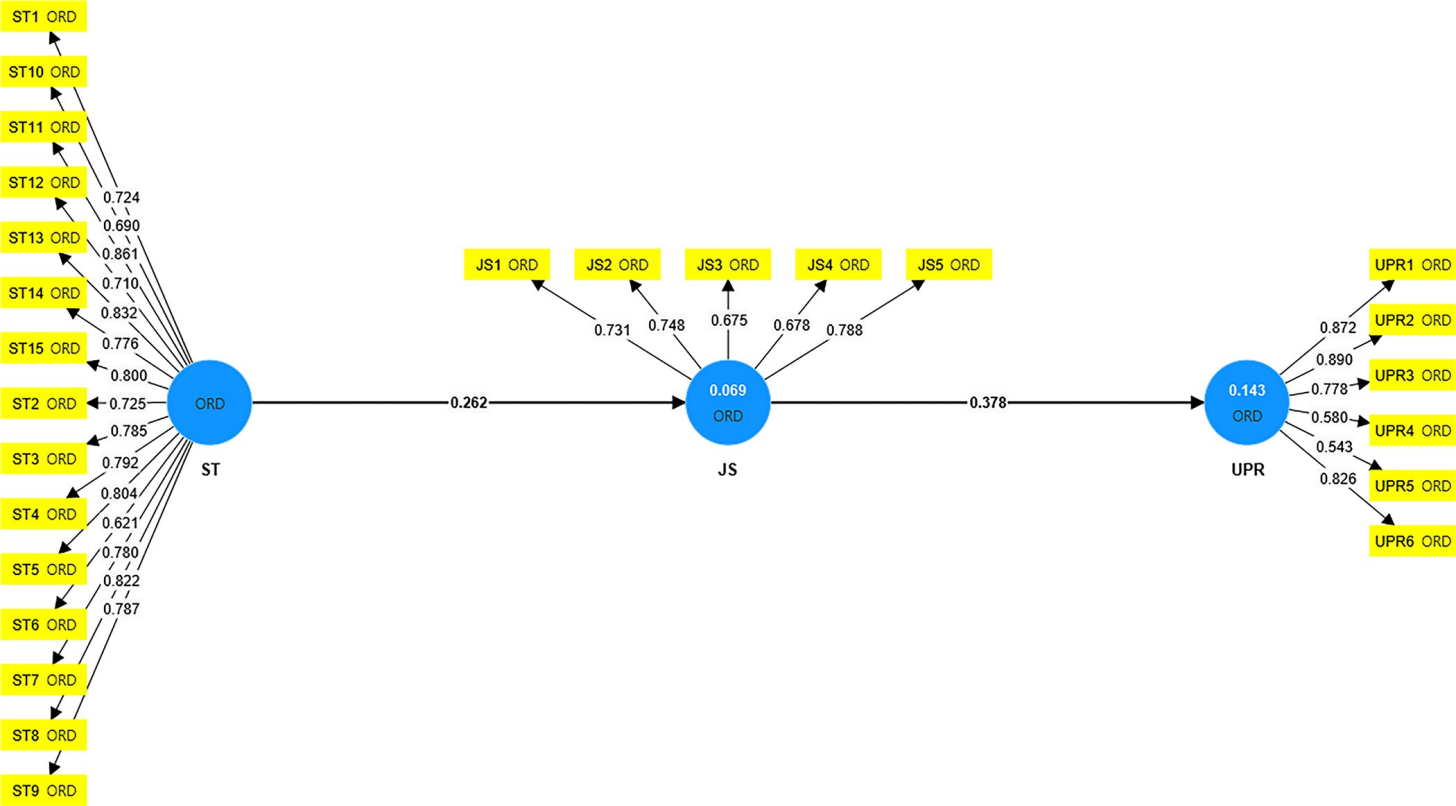
Measurement model.

The initial phase of PLS-SEM analysis involves assessing the measurement model, scrutinizing factor loadings, reliability, convergent validity, and discriminant validity of the constructs, as illustrated in Fig 2. The factor loadings of all items were examined, with most exceeding 0.600, except for two items (UPR4 and UPR5) which fell below this threshold. However, these items were retained as their exclusion did not significantly impact the average variance extracted (AVE) value of the corresponding construct [51]. Subsequent analysis indicated that Cronbach’s alpha, composite reliability (CR), and AVE values for all constructs surpassed the recommended thresholds, with values of α > 0.700, CR > 0.700, and AVE > 0.500 [51]. Further details can be found in Table 3.

**Table 3:** Reliability and convergent validity.

Constructs	Item	Loading	Alpha	rho_A	CR	AVE
Staff Training (ST)	ST1	0.724	0.773	0.777	0.847	0.526
	ST2	0.725				
	ST3	0.785				
	ST4	0.792				
	ST5	0.804				
	ST6	0.621				
	ST7	0.780				
	ST8	0.822				
	ST9	0.787				
	ST10	0.690				
	ST11	0.861				
	ST12	0.710				
	ST13	0.832				
	ST14	0.776				
	ST15	0.800				
Job Satisfaction (JS)	JS1	0.731	0.951	0.967	0.956	0.592
	JS2	0.748				
	JS3	0.675				
	JS4	0.678				
	JS5	0.788				
University Productivity (UPR)	UPR1	0.872	0.857	0.902	0.888	0.578
	UPR2	0.890				
	UPR3	0.778				
	UPR4	0.580				
	UPR5	0.543				
	UPR6	0.826				

Note: AVE, average variance extracted; CR, composite reliability. Source: Authors’ own findings.

Moreover, discriminant validity for all constructs was assessed via the Heterotrait-Monotrait (HTMT) method. Findings (Table 4) indicate that all HTMT ratios remained below the recommended threshold of 0.85 [52], confirming discriminant validity for all variables.

**Table 4:** Discriminant validity—HTMT ratio.

	JS	ST	UPR
Job Satisfaction (JS)			
Staff Training (ST)	0.279		
University Productivity (UPR)	0.425	0.748	

Source: Authors’ own findings.

### 4.5 Structural model

**Fig 3:**
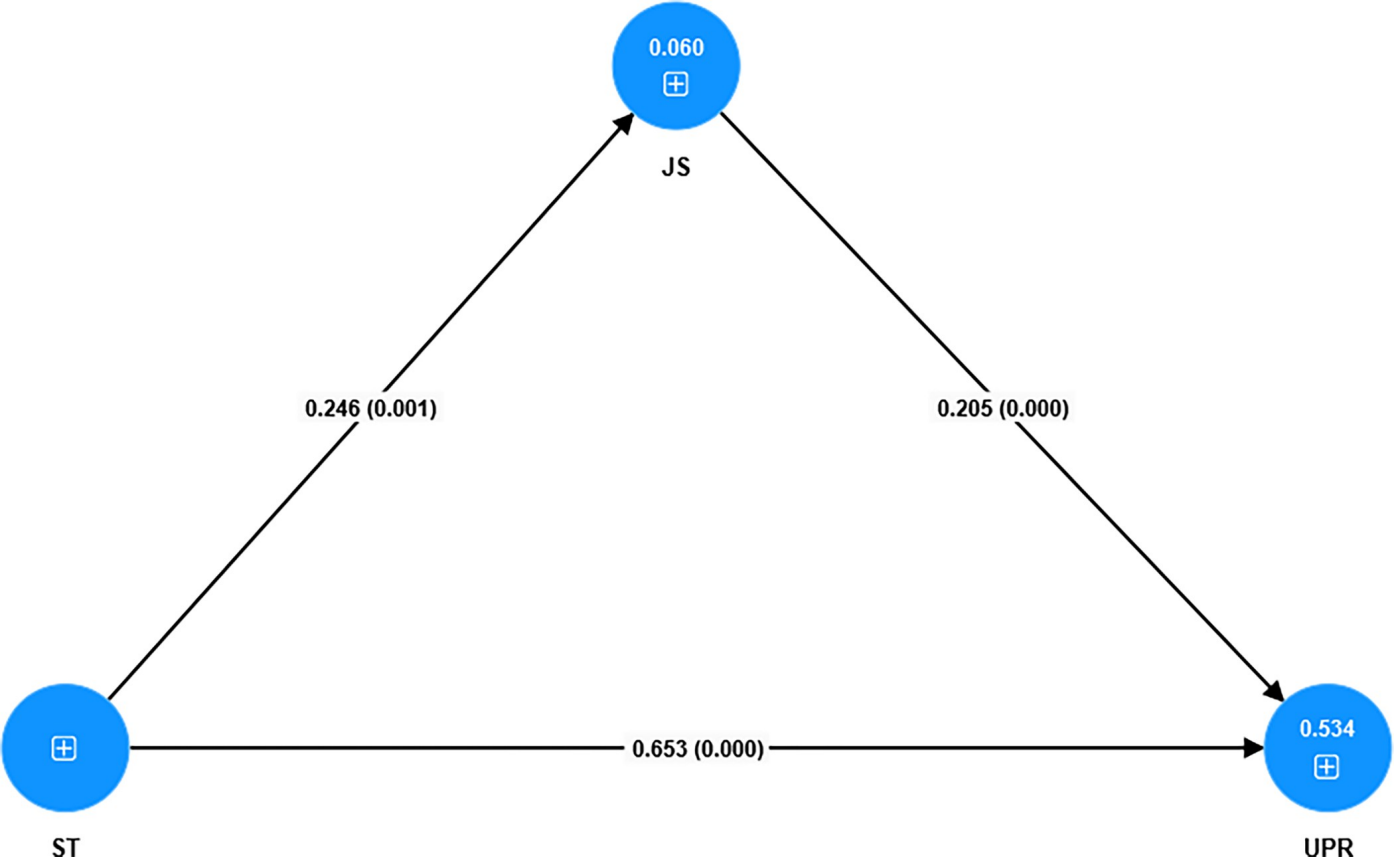
Structural model.

The structural model (see Fig 3), constituting the second stage of PLS-SEM analysis, was evaluated to test four hypotheses (see Table 5). Three direct effect hypotheses (H1–H3) were proposed and all were significant: H1: ST→UPR (β = 0.653, t = 13.386, p = 0.000), H2: ST→JS (β = 0.246, t = 3.102, p = 0.001), and H3: JS→UPR (β = 0.205, t = 3.418, p = 0.000), supporting H1, H2, and H3. Additionally, one hypothesis concerning indirect effects (mediation analysis) was proposed, indicating a significant mediating effect of job satisfaction: H4: ST→JS→UPR (β = 0.050, t = 1.987, p = 0.023), thereby supporting H4.

**Table 5:** Hypotheses testing results.

	Relationship	β	SD	t-value	p-value	Decision
H1	ST -> UPR	0.653	0.049	13.386	0.000	Supported
H2	ST -> JS	0.246	0.079	3.102	0.001	Supported
H3	JS -> UPR	0.205	0.060	3.418	0.000	Supported
H4	ST -> JS -> UPR	0.050	0.025	1.987	0.023	Supported

Source: Authors’ own findings.

## 5. Discussion and conclusion

### 5.1 Discussion on results

This study delves into how staff training on ISO 9001 requirements enhances job satisfaction among academic and administrative staff, ultimately leading to increased university productivity through continuous learning initiatives and knowledge management practices. Drawing upon the OLT, which suggests that organizations improve performance by acquiring, disseminating, and utilizing knowledge over time [19], the study aims to demonstrate the relevance of OLT theory in higher education in Pakistan. It provides a comprehensive framework for evaluating university productivity through organizational learning processes. From an OLT perspective, the study examines how staff training initiatives impact organizational capacities, focusing on the satisfaction of academic and administrative staff, and overall university productivity. OLT’s compatibility with quality management principles facilitates the exploration of the interplay between staff training, job satisfaction, and productivity within ISO 9001-certified universities in Pakistan.

The present study aimed to address three research questions within the higher education context of ISO 9001-certified universities in Pakistan. Firstly, it aimed to investigate the influence of staff training on both job satisfaction and university productivity. Secondly, it sought to explore the impact of job satisfaction on university productivity. Lastly, it aimed to examine the mediating role of job satisfaction in the relationship between staff training and university productivity. To achieve these objectives, this study tested four hypotheses (H1-H4). Among these, the first three hypotheses concerned direct relationships involving staff training, job satisfaction, and university productivity. The first hypothesis focused on the direct effects of staff training on university productivity and was found to be significant. This finding is supported by previous studies that have demonstrated the strong impact of staff training on academic staff productivity [17, 25], and librarians’ job performance [26].

The second hypothesis identified a significant impact of staff training on the job satisfaction of academic and administrative staff, consistent with prior research [16, 27, 28]. Similarly, the third hypothesis revealed a positive association between job satisfaction and university productivity, consistent with previous studies [28, 30–32]. The fourth hypothesis tested the mediation analysis of job satisfaction on staff training and university productivity, with results confirming its significance, supported by prior studies [28, 33–35].

### 5.2 Conclusion

This study examines the impact of training academic and administrative staff on their job satisfaction and university productivity, with a focus on ISO 9001-certified universities in Pakistan. Additionally, it explores how job satisfaction mediates the relationship between staff training and university productivity. The findings confirm the significance of these relationships. Using OLT, the study provides a comprehensive framework for evaluating university productivity through organizational learning processes. Specifically, it investigates how staff training initiatives affect organizational capacities, particularly the satisfaction of academic and administrative staff, and overall university productivity in ISO 9001-certified universities in Pakistan. The results underscore the pivotal role of training for academic and administrative staff in bolstering motivation and satisfaction, thus fostering increased productivity at both individual and institutional levels. This study enriches theoretical insights and practical implications by amalgamating the human capital perspective with ISO 9001 QMS in the higher education context.

## 6. Implications, limitations, and future directions

### 6.1 Theoretical implications

The present study contributes significantly to theoretical understanding by demonstrating the applicability of OLT within Pakistan’s higher education context, yielding several implications. Firstly, it validates the suitability of OLT within the context of ISO 9001-certified universities, affirming that organizations, including universities, can enhance productivity and job satisfaction through systematic staff training aligned with ISO 9001 requirements. Secondly, the study establishes a significant relationship between staff training on ISO 9001 requirements and job satisfaction, thereby augmenting the literature on QMS and their impact on employee attitudes and behaviors, consequently enhancing university productivity. Thirdly, the study illuminates the influence of ISO 9001 implementation, particularly through staff training, on various facets of university operations, thus enriching our understanding of the mechanisms by which quality management practices influence organizational outcomes. Finally, conducted within the specific context of Pakistani universities, the study offers insights into the application and adaptation of quality management practices, guided by international standards such as ISO 9001, across diverse cultural and organizational settings.

### 6.2 Practical implication

The study provides practical implications for various stakeholders. Firstly, it offers guidance for universities in Pakistan and similar contexts on effectively implementing ISO 9001 standards. Prioritizing staff training aligned with ISO 9001 requirements can enhance productivity and job satisfaction, facilitating successful certification and ongoing compliance. Secondly, organizations can use the study results to inform their human resource development strategies, as investing in staff training programs not only improves organizational performance but also boosts employee job satisfaction and retention. Thirdly, the study underscores the significance of continuous learning and improvement within universities. Administrators can devise strategic initiatives aimed at fostering a culture of organizational learning, where staff training acts as a catalyst for productivity enhancement and employee satisfaction. Finally, stakeholders such as policymakers and university administrators can leverage the study’s findings to shape policy formulation and decision-making processes concerning quality management practices in higher education. By prioritizing investments in staff training aligned with ISO 9001 standards, they can drive positive organizational outcomes and enhance the overall quality of education delivery.

### 6.3 Limitations and future directions

While providing valuable insights, the study is constrained by certain limitations. Notably, the absence of a sampling frame necessitated the utilization of a purposive sampling technique, thereby restricting participation to academic and administrative staff exclusively from seven ISO 9001-certified universities in Pakistan. Future research endeavors should prioritize broader stakeholder inclusion and explore the feasibility of employing random sampling techniques. Additionally, researchers should explore innovative approaches to better understand the factors influencing job satisfaction among academic and administrative staff, aiming to enhance productivity globally in ISO 9001-certified universities.

## Supporting information

S1 File Survey data. (XLSX)

S2 File (DOCX)
