# Supplementary material for: Impact of staff training on university productivity through job satisfaction: A study of ISO 9001- certified institutions
Source: PLoS One. 2024 Jul 9;19(7):e0306799. doi: 10.1371/journal.pone.0306799 (PMC11232989; doi:10.1371/journal.pone.0306799)
Supplement: S2 File — (DOCX) [file pone.0306799.s002.docx]

**Additional Information**

**Ethics Statement**

We, the authors, hereby declare that we have adhered to all ethical practices as delineated and discussed in the ethics approval letter throughout each stage of this study.

**Competing interests**

The authors have declared that no competing interests exist.

**Author Contributions**

The study concept was initiated by SI, with MH and SK contributing to the research design and aiding in data collection. SI conducted the data analysis and drafted the manuscript, while MH provided project supervision and reviewed the manuscript. SK verified the analytical methods and results. All three authors contributed equally to the discussion of results and the final manuscript.

**Funding**

This research did not receive any specific grant from funding agencies in the public, commercial, or not-for-profit sectors.

**Acknowledgments**

The authors would like to thank the reviewers and the editor for their insightful comments and suggestions.

**Data Availability Statement**

All relevant data are within the manuscript and its Supporting Information files.
